# Editorial: Antiviral monoclonal antibody therapies

**DOI:** 10.3389/fcimb.2024.1484448

**Published:** 2024-10-10

**Authors:** Catalina Florez, Denise Haslwanter, Rohit K. Jangra

**Affiliations:** ^1^ The Henry M. Jackson Foundation, Bethesda, MD, United States; ^2^ U.S Army Medical Research Institute of Infectious Diseases, Fort Detrick, Frederick, MD, United States; ^3^ Department of Microbiology and Immunology, Albert Einstein College of Medicine, Bronx, NY, United States; ^4^ Department of Microbiology and Immunology, Louisiana State University Health Sciences Center-Shreveport, Shreveport, LA, United States; ^5^ Center of Excellence for Emerging Viral Threats, Louisiana State University Health Sciences Center Shreveport, Shreveport, LA, United States; ^6^ Center for Applied Immunology and Pathological Processes, Louisiana State University Health Sciences Center Shreveport, Shreveport, LA, United States

**Keywords:** monoclonal Abs, antiviral, SARS-CoV-2, COVID, bispecific Ab

Monoclonal antibodies (mAbs) are potent antiviral therapeutics that offer advantages, including post-exposure protection and protection for immunocompromised individuals. They can be modified to express antigen-specific paratopes in mono-specific, bi-specific, and multi-specific formats. Most available mAbs are approved as cancer and auto-immune medications. However, the utility of antiviral mAbs has been underscored by their successful deployment against many viruses, such as Ebola virus, human respiratory syncytial virus, human cytomegalovirus, influenza virus, and SARS-CoV-2 (Pantaleo et al., 2022). This Research Topic, “Antiviral Monoclonal Antibody Therapies,” highlights recent advances in mAb development and their utility against rapidly evolving viral pathogens.

Production cost is a major hurdle that needs to be overcome to enable the widespread use of antiviral mAb treatments in low-income communities and countries. Shanmugaraj et al. demonstrate that *Nicotiana benthamiana* is a powerful cost-effective platform for producing mAbs in a scalable and affordable manner. This work describes the first report of SARS-CoV-2-specific mAbs produced in a plant expression system and characterizes their ability to retain neutralization properties *in vitro*.

Another important factor to consider with antiviral mAb therapeutics, is the emergence of escape variants and their recalcitrance to vaccines and mAb treatments. Two studies in this Research Topic describe different approaches to solving this problem. One highlights the utility of bispecific mAbs, which can be engineered to increase the breadth of mAbs (Yu et al.). The second delineates a simple and traditional hybridoma approach to mAb development and describes the development of a broadly neutralizing mouse anti-SARS-CoV-2-Spike mAb that neutralizes different SARS-CoV-2 variants (Wen et al.). Broadly reactive mAbs that target conserved epitopes are attractive targets because they are less likely to result in escape mutations due to the lower fitness of generated mutants.

Finally, Kim et al. performed a retrospective multicenter study in South Korea to analyze the effectiveness of Regdanvimab, a SARS-CoV-2-specific mAb, in a hospitalized COVID-19 cohort. They found that compared to the group that received supportive care, patients that received Regdanvimab had reduced progression to severe disease. This was true for the overall study population but not for the patients infected with the SARS-CoV-2 delta variant. This work highlights the potential clinical benefits of antiviral mAb treatments, but also demonstrates that treatment regimens need to be optimized as new variants emerge.

With this Research Topic of research articles, we aim to highlight recent advances in the development and production of antiviral mAbs. As emerging and re-emerging viral pathogens continue to cause outbreaks in human populations, a need for effective, flexible, and affordable antiviral treatments is ever-present.
