# Corrigendum: Editorial: Antiviral monoclonal antibody therapies

**DOI:** 10.3389/fcimb.2025.1558708

**Published:** 2025-02-03

**Authors:** Catalina Florez, Denise Haslwanter, Rohit K. Jangra

**Affiliations:** ^1^ The Henry M. Jackson Foundation, Bethesda, MD, United States; ^2^ U.S Army Medical Research Institute of Infectious Diseases, Fort Detrick, Frederick, MD, United States; ^3^ Department of Microbiology and Immunology, Albert Einstein College of Medicine, Bronx, NY, United States; ^4^ Department of Microbiology and Immunology, Louisiana State University Health Sciences Center-Shreveport, Shreveport, LA, United States; ^5^ Center of Excellence for Emerging Viral Threats, Louisiana State University Health Sciences Center Shreveport, Shreveport, LA, United States; ^6^ Center for Applied Immunology and Pathological Processes, Louisiana State University Health Sciences Center Shreveport, Shreveport, LA, United States

**Keywords:** monoclonal Abs, antiviral, SARS-CoV-2, COVID, bispecific Ab

In the published article, there was an error in the **Funding** statement. The NIH support for Rohit K Jangra (R.K.J.) is missing. The correct **Funding** statement appears below.

“This work was partly supported by grants (R21AI156482 and P20GM134974 to RJ) from the National Institutes of Health, USA.”

The authors apologize for this error and state that this does not change the scientific conclusions of the article in any way. The original article has been updated.

